# Interrelationships Among Individual Factors, Family Factors, and Quality of Life in Older Chinese Adults: Cross-Sectional Study Using Structural Equation Modeling

**DOI:** 10.2196/59818

**Published:** 2024-10-28

**Authors:** Yuting Wu, Cong Gong, Lifang Pi, Meixin Zheng, Weifang Liu, Yamei Wang

**Affiliations:** 1 Huanggang Hospital of Traditional Chinese Medicine Huanggang China; 2 State Key Laboratory of New Drug Discovery and Development for Major Diseases Gannan Medical University Ganzhou China; 3 Gannan Innovation and Translational Medicine Research Institute Gannan Medical University Ganzhou China; 4 Department of Cardiology, Renmin Hospital of Wuhan University Wuhan China; 5 Huangzhou District Healthcare Service Center Huanggang China

**Keywords:** quality of life, older adults, individual factor, family factor, structural equation modeling (SEM)

## Abstract

**Background:**

China's rapidly aging population necessitates effective strategies for ensuring older adults' quality of life (QOL). While individual factors (IF) and family factors (FF) are known to influence QOL, existing research often examines these factors in isolation or focuses on specific subpopulations, overlooking potential interactions and mediating pathways.

**Objective:**

This study aims to examine both direct and indirect pathways connecting IF and FF to older adults’ QOL, focusing on the mediating roles of health risks (HR) and health care service demand (HSD).

**Methods:**

This study uses structural equation modeling (SEM) to analyze cross-sectional data from 8600 older participants in the 2015 China Health and Retirement Longitudinal Study (CHARLS), a nationally representative study using a multistage probability proportional to size sampling method.

**Results:**

Among the 8600 participants, the majority (5586/8502, 65.7%) were aged 60-70 years, with a near-equal distribution of males and females at around 50%. The average PCS score was 76.77, while the MCS score averaged 59.70. Both IF (β=0.165, *P*<.001) and FF (β=0.189, *P*<.001) had a direct positive effect on QOL. Furthermore, the indirect effects of IF (β=0.186, *P*<.001) and FF (β=0.211, *P*<.001) through HR and HSD were also significant. In the direct model, IF and FF had a greater impact on MCS (β=0.841) than on PCS (β=0.639). However, after including the 2 mediating factors, HR and HSD, the influence of IF and FF on MCS (β=0.739) became consistent with that on PCS (β=0.728). Subgroup analyses revealed that the direct effect of IF on QOL was significant in the 60-70 age group (β=0.151, *P*<.001) but not in those over 70 years old (β=0.122, *P*=.074). Comorbidity status significantly influenced the pathway from HR to HSD, with older adults having 2 or more chronic diseases (β=0.363) showing a greater impact compared to those with fewer than 2 chronic diseases (β=0.358).

**Conclusions:**

Both IF (education, per capita disposable income, and endowment insurance) and FF (satisfaction with a spouse and children) directly impact the QOL in older people. Meanwhile, IF and FF have equal influence on QOL through the mediating role of HR and HSD. Recognizing the interplay among these factors is crucial for targeted interventions to enhance the well-being of older adults in China.

## Introduction

Since China transitioned into an aging society around 2000, the aging process has accelerated steadily [1]. It is projected that by 2040, the population aged 60 years and older in China will reach 402 million, with an aging rate exceeding 28%, marking the country's transition into a deeply aging society [2]. The increased number of older adults will inevitably lead to a substantial rise in national financial pressure and family care burden [3,4]. Ensuring a dignified and high quality of life (QOL) for older adults during their later years has evolved into a pivotal focal point within the sphere of social development.

QOL refers to an individual's overall well-being and satisfaction with various aspects of their life [5]. It is directly correlated with overall health outcomes, health care use, and the effectiveness of health interventions. QOL serves as a pivotal benchmark illuminating the living standards of older adults, forming the cornerstone for the implementation of strategies promoting healthy and active aging. The health status of older individuals is a key determinant of their overall physical and mental well-being. While considerable research has been devoted to exploring the risk factors affecting the health status of older adults [6-8], comprehensive studies integrating various sociodemographic factors, such as individual and family factors, which broadly impact their QOL, remain limited. Individual factors (IFs) include socioeconomic and demographic characteristics [9]. In this study, IF is represented by education level [10], per capita disposable income [11], and endowment insurance [12]. Family factors (FFs) include interactions among family members and the broader family environments [13]. In this study, FF is represented by measures of spouse satisfaction [14] and children satisfaction [15].

Existing literature has extensively examined the impact of various individual and family factors on the QOL of older adults, highlighting the importance of maintaining healthy lifestyles and fostering strong social connections to enhance QOL in later life [16,17]. For example, studies by Zhang et al [18] in Shanxi, China, and Chen et al [19] in Hainan, China, delved into the influence of socioeconomic status, health behaviors, and family relationships on the QOL of older adults. However, these studies often either focus on the impact of a single factor or target subgroups with specific characteristics, such as empty nesters and the oldest-old population. This overlooks the potential mutual interactions between variables and the interactive effects on outcomes, potentially resulting in partial and nongeneralizable research conclusions.

Furthermore, previous studies indicated that IFs, such as education and income, impact dietary habits, smoking and alcohol consumption, physical activity levels [20,21], and access to health insurance in older adults [22]. Similarly, FFs, such as marital status and parent-child relationships, affect health risks (HR) and mental well-being in older adults [23,24]. In turn, HR can influence health care service demand (HSD) [25]. Both HR and HSD are known to be associated with QOL [26]. Nevertheless, only a few studies have addressed the potential mediating role of HR and HSD in the relationship between individual or familial factors and QOL. For example, Zhang et al [18] demonstrated the indirect effects of socioeconomic status and family relationships on QOL through health behaviors among empty nesters. Similarly, studies emphasized the importance of addressing health-related issues and improving access to health care services to enhance QOL among older adults in Zhejiang province and Shanghai [27,28]. However, these studies focus on specific regions or populations, and most studies rely on cross-sectional designs, hindering the establishment of causal relationships. There is a need for more comprehensive frameworks that integrate a broader range of personal, familial, and environmental factors and examine their complex interactions and potential mediating roles to provide a holistic understanding of QOL among older adults.

This study uses structural equation modeling (SEM) to analyze the direct and indirect effects of individual and family factors on QOL among individuals aged 60 years and older that cover the entire scope of China. We consider the mediating roles of HR and HSD in the relationship between individual or family factors and QOL. By examining these complex relationships, this study aims to provide valuable insights for policy making and interventions to improve the well-being of older adults in China.

## Methods

### Data Source and Participants

This study used data from the China Health and Retirement Longitudinal Study (CHARLS), the details of which have been documented previously [29]. Briefly, the CHARLS project aims to gather high-quality microdata representing households and individuals aged 45 years and older in China, promoting interdisciplinary aging research. The national baseline survey of CHARLS was conducted in 2011 using a multistage probability proportional to size sampling method. The sample encompassed 450 villages, 150 counties, and 28 provinces, comprising over 17,000 individuals from approximately 10,000 households. CHARLS is an ongoing survey, with assessments conducted every 2-3 years. Participants were interviewed face-to-face in their homes using computer-assisted personal interviewing technology. The survey comprehensively covered basic demographic information of respondents and their families, intrafamily transfer payments, health status, medical care and insurance, employment, income, expenditure, assets, and more. Additionally, CHARLS included 13 physical measurements and collected blood samples. To date, CHARLS has released data from 5 waves: the national baseline survey (Wave 1, 2011), first follow-up survey (Wave 2, 2013), second follow-up survey (Wave 3, 2015), third follow-up survey (Wave 4, 2018), and fourth follow-up survey (Wave 5, 2020) [30]. The CHARLS datasets can be downloaded at the CHARLS home page [30]. The CHARLS survey project received approval from the Biomedical Ethics Committee of Peking University, and all participants have provided informed consent.

**Figure 1:**
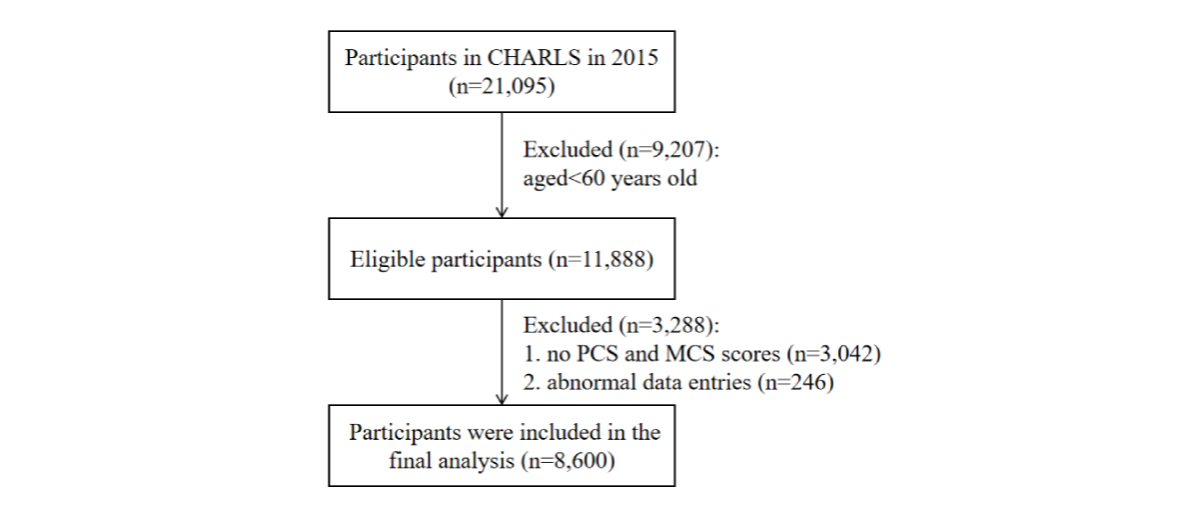
The flowchart of participants through the study. CHARLS: China Health and Retirement Longitudinal Study; PCS: physical component summary; MCS: mental component summary.

This study used data from the third wave of the CHARLS, collected in 2015, with a sample size of 21,095. We excluded 12,495 individuals due to (1) age <60 years (n=9207); (2) missing information on physical component summary (PCS) and mental component summary (MCS) scores (n=3042); and (3) abnormal data entries (n=246), resulting in a final analytic sample of 8600 participants for this cross-sectional analysis. The detailed flowchart of the sample selection process is shown in Figure 1.

### Conceptual Framework

**Figure 2:**
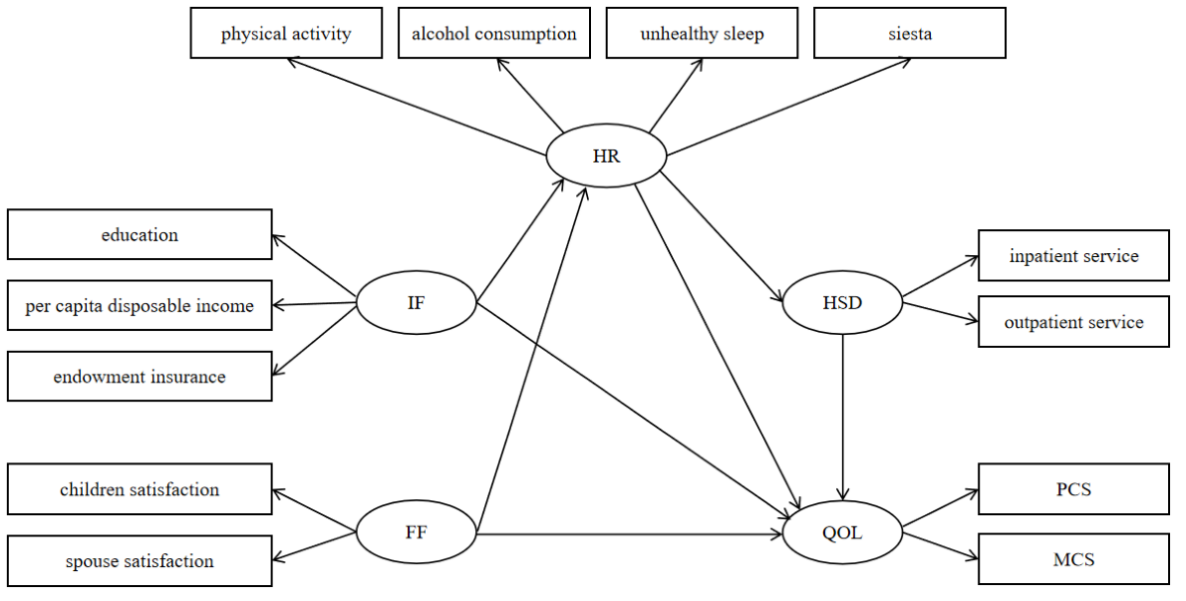
Research framework. IF: individual factors; FF: family factors; HR: health risk; HSD: health services demand; PCS: physical component summary; MCS: mental component summary; QOL: quality of life.

Building upon a review of the literature [16,26-30] and informed by medical context, the core components of the conceptual framework in this study include: (1) IF is associated with education [10], per capita disposable income [11], and endowment insurance [12], which directly influence QOL. (2) FF is associated with spouse satisfaction [14] and children satisfaction [15], which directly influence QOL. (3) Individual and family factors indirectly influenced QOL via HR. The reason is that health plays a pivotal role in overall well-being [18]. IFs, such as education and income, also impact dietary habits, smoking and alcohol consumption, and physical activity levels in older adults [31]. Similarly, FFs, such as marital status and parent-child relationships, affect health risks and mental well-being in older adults [23,24]. HR encompasses physical activity, alcohol consumption, unhealthy sleep, and siesta. (4) IF and FF indirectly influenced QOL via HR and HSD. HSD involves both inpatient service and outpatient service. Previous studies have found that HR can influence HSD [25]. Both HR and HSD are known to be associated with QOL [26]. (5) Assessing the QOL through the metrics of PCS and MCS, which is accessed by Short Form 36 (SF-36) [32]. The multiple pathways from IF and FF to QOL and the statistical analysis strategy are shown in Figure 2.

### Measures

This study introduced a novel scale construction from variables derived from both SF-36 and the CHARLS questionnaire to measure QOL in older Chinese adults. This scale's development emanated from the 8 dimensions within the SF-36, while we curated pertinent CHARLS variables to appraise these dimensions, encompassing physical functioning (PF), role-physical (RP), bodily pain (BP), general health (GH), vitality, social functioning (SF), role-emotional (RE), and mental health (MH). PF, RP, BP, and GH were computed as PCS, while vitality, SF, RE, and MH were computed as MCS (Multimedia Appendix 1). Each dimension score was converted to a range from 0 to 100, with a high score indicating better levels of functioning. The SF-36 score was obtained from the average of the 8 dimensions. Consequently, higher SF-36 scores indicate better QOL [32].

Drawing on methodologies applied in pertinent literature, the development of this scale mirrored approaches using Cronbach α coefficient to assess reliability between SF-36 and CHARLS variables. Overall, the scale demonstrated robust reliability across dimensions, registering α values exceeding 0.6 for all except vitality (α=0.34) [33]. At both the item (I-CVI) and scale (S-CVI) levels, the content validity index (CVI) was assessed, with I-CVI surpassing 0.83 and an S-CVI reaching 0.94 [33]. These findings suggest that the scale exhibited commendable reliability and validity and has already been used in related research [34].

Demographic characteristics included sex (male or female) and age groups (60-70 years and 70 years or older). We defined comorbidity as the presence of ≥2 chronic diseases [35]. IF was accessed by 3 variables: education [10], per capita disposable income [11], and endowment insurance [12]. The specific definition of each variable is added in Multimedia Appendix 2. It is important to note that per capita disposable income, presented as a continuous variable, was extracted from the 2015 Gotohui data [36], takes into account deductions, such as personal income tax, social insurance contributions, and removes essential living expenses to measure individuals' actual disposable income within an economy [37]. The following 2 variables accessed FF: spouse satisfaction [14] and children satisfaction [15]. HR was accessed by 4 variables: physical activity [38], alcohol consumption [39], unhealthy sleep, and siesta [40,41]. Thereby, HSD was accessed using 2 variables: inpatient service and outpatient service [42].

### SEM and the Theory of Inferred Causation

First, SEM allows for constructing latent variables using other observable variables and models causal relationships between latent and observed variables [43]. Second, within SEM, causal paths are explicitly represented as directed arrows between variables in a path diagram [44]. These paths represent hypothesized causal relationships based on theory or prior research [44]. Finally, SEM evaluates how well the specified model fits the observed data to evaluate the validity of hypothesized causal relationships [45]. A well-fitting model indicates that the hypothesized causal relationship model can effectively explain or predict the observed variability in the data [44]. Additionally, the magnitude of each path parameter signifies the strength and direction of the causal paths between variables [43]. In cases of poor model fit, researchers can refine models based on empirical data and theoretical insights, enhancing the accuracy of causal inferences [44].

### Statistical Analysis

Continuous variables are presented as mean and SD, and categorical variables are presented as frequencies and percentages. Scores of QOL are presented as mean and SD. Pearson correlation analysis is used to examine the correlations among the IF, FF, HR, HSD, and QOL variables. The missing data were imputed using nonparametric missing value imputation based on the random forest procedure in R. Detailed missing rates for the essential factors used in this study are provided in Multimedia Appendix 3.

To verify the suitability of the SF-36 scale based on the CHARLS database for the construction of SEM, we performed confirmatory factor analysis (CFA) [46]. In CFA, CR is used to demonstrate composite reliability, and average variance extracted (AVE) is used to establish the convergent validity of the model. The model exhibits strong internal consistency and convergent validity, as indicated by the CR exceeding 0.7 and the AVE surpassing 0.5, and the AVE between 0.36 and 0.5 is also acceptable [47].

SEM was used to ascertain the direct and indirect effects among outcome, observed, and latent variables. Among them, the observed variable refers to a variable that can be directly observed with specific values. In contrast, the latent variable is a variable that cannot be directly observed and requires inference through other variables. The total effect represents the sum of direct and indirect effects, mathematically expressed as follows [48]: c = c′ + ab, where c = total effect, c′ = direct effect, ab = indirect effect. Subsequently, bias-corrected bootstrapping (using 2000 bootstrap samples) was used to gauge the statistical significance of both direct and indirect effects within each pathway of the SEM. Various modification indices were used to refine and adjust the model, aiming to achieve the optimal fit [49], including the comparative fit index (CFI), incremental fit index (IFI), standardized root-mean-square residual (SRMR), goodness of fit index (GFI), adjusted goodness of fit index (AGFI), and the root-mean-square error of approximation (RMSEA). The RMSEA and SRMR ≤ 0.08, CFI, IFI, GFI, and AGFI > 0.90 indicated an acceptable model [50]. Due to the sensitivity of chi-square values to large sample sizes, they were excluded from the analysis [50]. Main effect values are presented by β, representing standardized regression coefficients. *P*< .05 indicated statistical significance. In addition, given that approximately 50% of the data on physical activity were missing, we conducted an additional sensitivity analysis to assess the robustness of the results by excluding participants with missing physical activity data.

Finally, we conducted subgroup analyses to examine the applicability of the model across gender, age, and comorbidity groups and to assess whether there were significant differences in the β values of different paths among the subgroups. In subgroup analyses, an absolute critical ratio for a difference of more than 1.96 was considered to indicate a significant difference between the groups [51]. *P*<.05 for β indicates its statistical significance. All data were analyzed using the statistical software IBM SPSS Statistics (version 26.0; IBM Corp), IBM SPSS Amos (version 26.0), and R (version 4.3.2; R Foundation for Statistical Computing).

### Ethical Considerations

This study used secondary data from CHARLS. The agency responsible for the survey is Peking University.

## Results

### Participant Characteristics

Table 1 summarizes the results of descriptive statistics. Among the 8600 samples, the gender distribution was nearly equal. Participants aged 60-70 years outnumbered those aged over 70 years (n=5586 vs n=2916). Concerning comorbidity, a modest proportion (3171/8600, 36.9%) of the participants had more than 2 chronic diseases. Most participants reported their educational attainment as less than the secondary level and did not have endowment insurance. The mean annual per capita disposable income in the study population was ¥ 17,715.97 (SD ¥ 10,131.80; US $2852.27, per the 2015 average annual exchange rate). A significant number of participants reported satisfaction with their spouses and children.

**Table 1:** Descriptive statistics of the variables at baseline.

Variables			Overall, n (%)
**Age (years), (n=8502)**			
	60-70	5586 (65.7)	
	≥70	2916 (34.3)	
**Gender, (n=8599)**			
	Male	4297 (50.0)	
	Female	4302 (50.0)	
**Comorbidity, (n=8600)**			
	Fewer than 2 chronic diseases	5429 (63.1)	
	Having 2 or more chronic diseases	3171 (36.9)	
**Education, (n=8598)**			
	Below secondary education	7912 (92.0)	
	High school and vocational training	534 (6.2)	
	Higher education	152 (1.8)	
**Endowment insurance, (n=8550)**			
	Having endowment insurance	1097 (12.8)	
	No endowment insurance	7453 (87.2)	
**Spouse satisfaction, (n=6939)**			
	Satisfied with spouse	6355 (91.6)	
	Dissatisfied with spouse	584 (8.4)	
**Children satisfaction, (n=8494)**			
	Satisfied with children	8080 (95.1)	
	Dissatisfied with children	414 (4.9)	
**Alcohol consumption, (n=8596)**			
	Drinking alcoholic beverages in the past year	6403 (74.5)	
	Not drinking alcoholic beverages in the past year	2193 (25.5)	
**Physical activity, (n=4259)**			
	Engaging in any physical activity in the past week	2990 (70.2)	
	Have not engaged in any strenuous physical activity in the past week	1269 (29.8)	
**Unhealthy sleep, (n=7915)**			
	Sleep less than 5 hours or more than 9 hours per night	3084 (39.0)	
	Sleep duration is within 6-8 hours	4831 (61.0)	
**Siesta, (n=7982)**			
	Having a habit of taking midday naps	4606 (57.7)	
	Absence of the habit of taking midday naps	3376 (42.3)	
**Outpatient services, (n=8588)**			
	Received outpatient or home care services in the past month	1767 (20.6)	
	Not attending outpatient or home health services in the past month	6821 (79.4)	
**Inpatient services, (n=8588)**			
	Inpatient service use in the past year	1436 (16.7)	
	No inpatient service use in the past year	7152 (83.3)	

In terms of lifestyle factors, most participants engaged in physical activity and reported alcohol consumption in the past year. Healthy sleep patterns were prevalent. More than half of the individuals indicated good sleep quality and had regular siestas. Health care use patterns indicated that only a minority of the participants had received inpatient services in the past year and used outpatient or home care services within the past month during the baseline period.

### Status Quo of the Older Chinese Adults’ QOL

Table 2 displays the total scores and dimensional scores of SF-36. Among the 8600 participants, the PCS score averaged 76.77, and the MCS score averaged 59.70. Among the 4 dimensions of PCS, role physical obtained the highest score, followed by bodily pain and physical functioning. The general health dimension had the lowest score. In the realm of MCS, vitality, role emotional, and mental health scores were similar, all above 70.00, while social functioning scored lower.

**Table 2:** Scores for quality of life (n=8600).

Dimensions	Scores, mean (SD)
PCS^a^	76.77 (14.50)
PF^b^	85.54 (17.61)
RP^c^	94.99 (13.70)
BP^d^	87.90 (22.80)
GH^e^	38.66 (26.08)
MCS^f^	59.70 (17.83)
Vitality	77.29 (27.05)
SF^g^	13.18 (13.63)
RE^h^	72.35 (31.66)
MH^i^	75.97 (23.51)

^a^PCS: physical component summary.

^b^PF: physical functioning.

^c^RP: role-physical.

^d^BP: bodily pain.

^e^GH: general health.

^f^MCS: mental component summary.

^g^SF: social functioning.

^h^RE: role-emotional.

^i^MH: mental health.

### Correlation Between Variables

The correlation analysis results between IF, FF, HR, HSD, and QOL variables are detailed in Multimedia Appendix 4. The results indicate that the observed IF, FF, HR, and HSD variables significantly correlate with the outcome indicators. Meantime, the significant correlation coefficients between each observed variable ranged from –0.007 to 0.307, which did not meet the standard of strong correlation [52].

### CFA Results

The detailed results of the CFA performed to assess the suitability of the SF-36 scale based on the CHARLS database for the construction of SEM are presented in Table 3. According to the results, the standardized regression coefficients, ranging from 0.132 to 0.839, were statistically significant in the 2-factor model. The CR and AVE of the PCS and MCS were within the acceptable range. These findings indicate that the model exhibited excellent reliability and validity, rendering it suitable for SEM analysis. The fit indexes for the entire sample model, as shown in Table 4, suggest a good model fit.

**Table 3:** Standardized regression coefficient, composite reliability, and convergent validity of the CFA^a^.

Path		β^b^	*P* value	CR^c^	AVE^d^
**PCS** ^e^			<.001	0.726	0.412
	PCS→physical function	0.831			
	PCS→role-physical	0.692			
	PCS→bodily pain	0.526			
	PCS→general health	0.448			
**MCS^f^**			<.001	0.704	0.419
	MCS→vitality	0.650			
	MCS→social functioning	0.132			
	MCS→role-emotional	0.738			
	MCS→mental health	0.839			

^a^CFA: confirmatory factor analysis.

^b^β: standardized regression coefficient.

^c^CR: composite reliability.

^d^AVE: average variance extracted.

^e^PCS: physical component summary.

^f^MCS:mental component summary.

**Table 4:** Model-fit index of CFA^a^ and SEM^b^ (n=8600).

Inspected Fit Indices	Acceptable Fit	CFA Fit Indices	Direct SEM Fit Indices	Multiple intermediary SEM Fit Indices
SRMR^c^	≤0.08	0.060	0.017	0.031
RMSEA^d^	≤0.08	0.091	0.018	0.038
GFI^e^	>0.9	0.958	0.998	0.985
AGFI^f^	>0.9	0.921	0.996	0.977
CFI^g^	>0.9	0.926	0.995	0.917
IFI^h^	>0.9	0.926	0.995	0.917

^a^CFA: confirmatory factor analysis.

^b^SEM: structural equation model.

^c^SRMR: standard root-mean-square residual.

^d^RMSEA: root-mean-square error of approximation.

^e^GFI: goodness of fit index.

^f^AGFI: adjusted goodness of fit index.

^g^CFI: comparative fit index.

^h^IFI: incremental fit index.

### SEM Results

#### Direct Effects Model

**Figure 3:**
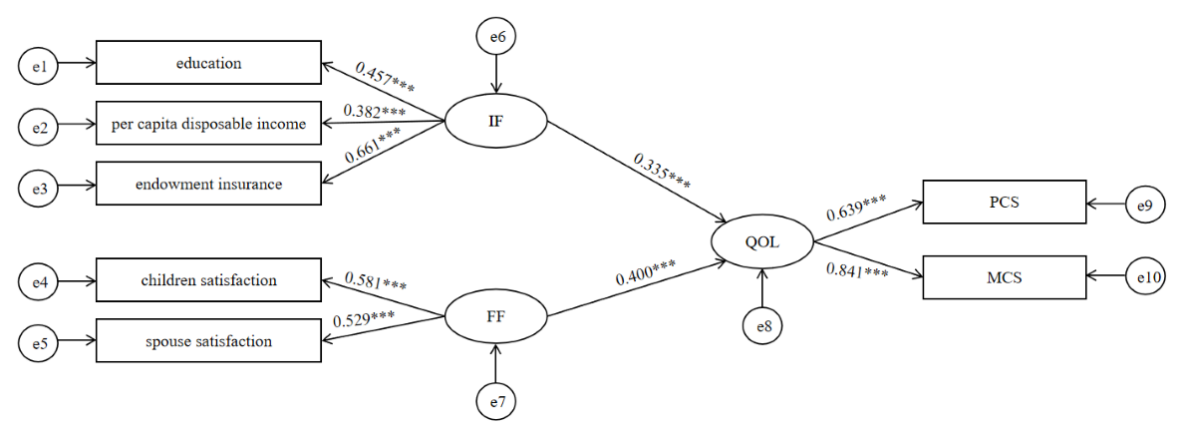
The direct effects of IF and FF on QOL. IF: individual factors; FF: family factors; PCS: physical component summary; MCS: mental component summary; QOL: quality of life.

Before analyzing the intermediary effects model, we used a total direct model to assess the effect of IF and FF on the QOL (Figure 3). The fit index of the direct model is acceptable (Table 4). In the direct effects model of individual and family factors on the QOL of older adults, IF and FF have significant positive direct effects on MCS and PCS, thereby promoting an improvement in QOL. It is worth noting that the impact of FF on QOL is greater than that of IF (0.400 vs 0.335; Figure 3). Moreover, the β value of endowment insurance to QOL in IF is much higher than the β values of education and per capita disposable income (0.661 vs 0.457 and 0.382). Additionally, in QOL, the β value of MCS is greater than the β value of PCS (0.841 vs 0.639), indicating that the direct impact of individual and family factors on MCS is greater than that on PCS (Figure 3).

#### Multiple Indirect Effects Model

**Figure 4:**
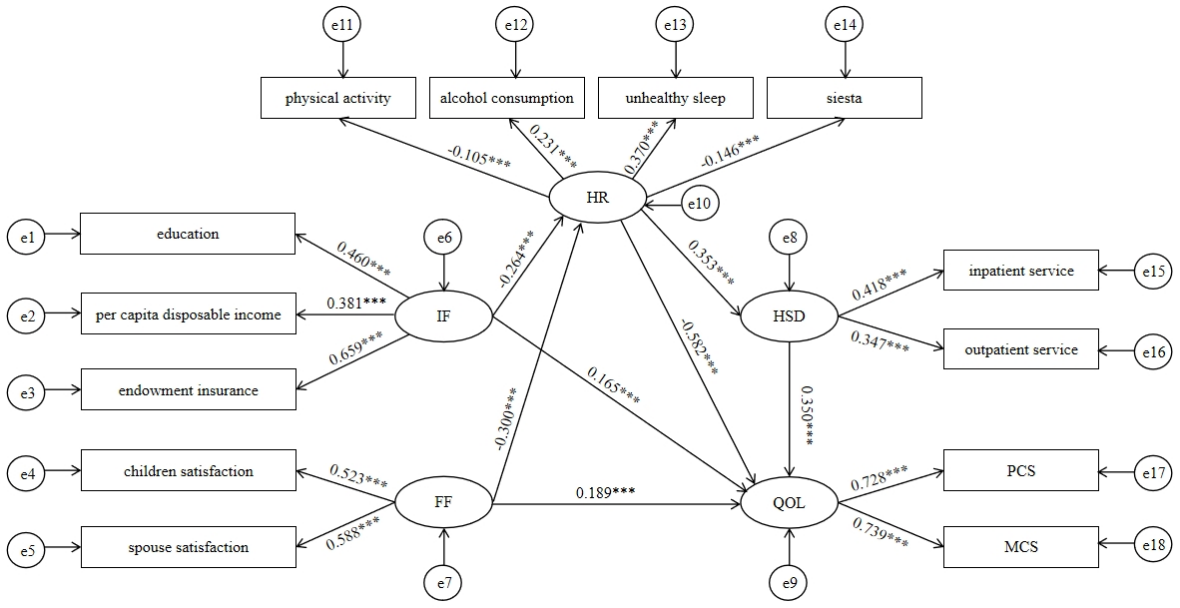
Multiple mediation model between IF, FF, and QOL with HR and HSD as mediators. IF: individual factors; FF: family factors; HR: health risk; HSD: health services demand; PCS: physical component summary; MCS: mental component summary; QOL: quality of life.

Second, we formulated a multiple intermediary effects model to explore how IF and FF influence the QOL among older adults through mediators—specifically, HR and HSD (Figure 4 and Multimedia Appendix 5). Table 4 illustrates the detailed information on model fit indices, revealing an acceptable fit for the multiple mediation mode. Table 5 further presents the total, direct, and indirect effects of IF and FF on QOL and their corresponding 95% CI.

**Table 5:** Standardized total, direct, and indirect effects of the variables on QOL^a^.

Mediation Effect	β^b^	Lower^c^	Upper^d^	Ratio of indirect to total effect, %	Ratio of direct to total effect, %
IF^e^→HR^f^→QOL	0.154	0.104	0.219	44	—^g^
IF→HR→HSD^h^→QOL	0.033	0.023	0.048	9	—
Total indirect effects: IF→QOL	0.186	0.128	0.254	53	—
Direct Effects: IF→QOL	0.165	0.100	0.219	—	47
Total Effects: IF→QOL	0.351	0.321	0.381	—	—
FF^i^→HR→QOL	0.174	0.119	0.240	44	—
FF→HR→HSD→QOL	0.037	0.025	0.054	9	—
Total Indirect Effects: FF→QOL	0.211	0.150	0.282	53	—
Direct Effects: FF→QOL	0.189	0.112	0.258	—	47
Totally Effects: FF→QOL	0.400	0.359	0.443	—	—

^a^QOL: quality of life.

^b^β: standardized regression weights.

^c^Lower: lower limit of confidence interval.

^d^Upper: upper limit of confidence interval.

^e^IF: individual factors.

^f^HR: health risk.

^g^Not applicable.

^h^HSD: health services demand.

^i^FF: family factors.

Specifically, the indirect effects of IF and FF on QOL mediated by HR accounted for a similar proportion of the total effect, both approximately 44%. Similarly, the proportions of the total effect attributed to the indirect effects mediated by HR and HSD were also comparable, around 9%. The CIs corresponding to each mediation path did not include 0, indicating that the multiple mediating effects between IF, FF, and QOL were valid. Additionally, the β values for PCS and MCS were similar in the multiple indirect effects model compared with the direct model (0.728 and 0.739 vs 0.639 and 0.841). This suggests that the impacts of IF and FF on PCS were strengthened following the inclusion of the mediation effect, gradually aligning with the effect observed for MCS. In conclusion, the indirect effects of IF and FF on QOL play equally significant roles as the direct effects, and the indirect effect through HR surpasses that through both HR and HSD. This indicates the pivotal role of HR in mediating the impact of IF and FF on QOL.

#### Subgroup Analyses

The model fit indices for subgroup analysis are presented in Multimedia Appendix 6. All fit indices across different subgroups fall within the acceptable range, indicating that the model is equally applicable across different genders, age groups, and comorbidities.

The detailed results of the subgroup analysis are presented in Table 6. In the gender subgroup analysis, all path coefficients (β values) are significant, and the absolute values of critical ratios for difference are all below 1.96, indicating no significant differences in the paths across different gender groups. In the age subgroup analysis, the absolute values of critical ratios for difference are all less than 1.96, indicating no significant differences in other paths across different age groups. It is worth noting that in terms of the effect of IF on QOL, the β value in the 60-70 age group was significant, and the β value in the group older than 70 years was not significant, indicating that for older adults aged 60-70 years, IF has a direct impact on QOL, but for older adults older than 70 years, IF has no direct effect on QOL. In the comorbidity subgroup analysis, the absolute value of the critical ratio for difference (–3.011) for the path from HR to HSD is greater than 1.96, indicating a significant difference in comorbidity for the HR to HSD path. Specifically, the effect of HR on HSD for older adults with 2 or more chronic diseases (0.363) is greater than for those with fewer than 2 chronic diseases (0.358). However, the absolute values of critical ratios for difference for other paths are all less than 1.96, demonstrating no differences in β values among paths due to comorbidity status.

#### Sensitivity Analysis

Due to the high missingness in physical activity data, we conducted an additional sensitivity analysis to evaluate the robustness of our findings. A total of 4259 participants with physical activity data were included in the analysis. The results indicate that the model fit indices are within acceptable ranges, and all path coefficients of the structural model are significant, suggesting that the missing physical activity data have minimal impact on the overall robustness of the multiple indirect effects model. Detailed results can be found in Multimedia Appendices 7 and 8.

**Table 6:** Standardized regression coefficients (β) with *P* values for the components of subgroup analyses.

Path	Gender				Age				Comorbidity		
	Male	Female	Critical ratios for difference	60-70 years old		≥70 years old	Critical ratios for difference	≥2 chronic diseases		<2 chronic diseases	Critical ratios for difference
IF^a^→HR	–0.215^b^	–0.154^b^	–0.903	–0.269^b^		–0.404^b^	0.100	–0.235^b^		–0.288^b^	0.221
FF^c^→HR	–0.251^b^	–0.293^b^	0.620	–0.283^b^		–0.326^b^	–0.006	–0.232^b^		–0.344^b^	–1.258
HR^d^→HSD^e^	0.401^b^	0.353^b^	–1.376	0.368^b^		0.175^f^	–0.319	0.363^b^		0.358^b^	–3.011
IF→QOL^g^	0.235^b^	0.170^b^	1.090	0.151^b^		0.122 (0.074)	1.225	0.194^b^		0.131^h^	0.112
FF→QOL	0.225^b^	0.207^b^	–0.413	0.161^b^		0.243^b^	–0.852	0.272^b^		0.118^f^	0.530
HR→QOL	–0.469^b^	–0.519^b^	–1.258	–0.631^b^		–0.514^b^	–0.012	–0.535^b^		–0.639^b^	–0.321
HSD→QOL	–0.381^b^	–0.366^b^	–0.460	–0.354^b^		–0.386^b^	–1.040	–0.329^b^		–0.357^b^	–1.263

^a^IF: individual factors.

^b^*P*<.001.

^c^FF: family factors.

^d^HR: health risk.

^e^HSD: health services demand.

^f^*P*<.05.

^g^QOL: quality of life.

^h^*P*<.01.

## Discussion

### Principal Findings

To our knowledge, this study is the first to comprehensively assess the impact of IF and FF on QOL in a representative Chinese population aged 60 years and older. We used latent variable modeling to estimate the combined effects of various factors on QOL among older adults. Additionally, we investigated potential mediating pathways beyond the direct effects of IF and FF on QOL. Our findings indicate that both IF and FF have a positive direct impact on QOL. FF showed a greater influence on QOL than IF. Notably, endowment insurance emerged as a significant determinant of QOL among IF. Moreover, indirect effects mediated by HR and HSD were found equally significant as direct effects, with HR playing a pivotal role among the mediators. While individual and family factors had a notably greater direct impact on MCS compared to PCS, their influence converged when considering mediators. Furthermore, we identified differential effects among subgroups with different age and comorbidity status.

Our study aligns with previous studies identifying factors influencing QOL, including education, income, and family relationships [19,28]. However, previous studies often focused on the impact of individual or family factors in isolation or on specific subgroups, such as solitary older adults [27] and community-dwelling older adult populations [53]. In contrast, our study uses structural equation modeling to consider the interactions between variables and their mutual influences on outcomes. Additionally, our study encompasses a nationwide sample of older adults aged 60 years and above in China, enhancing the representativeness and generalizability of the findings. Furthermore, we compared the combined effects of individual and family factors on QOL and found that FF has a greater direct influence than IF. Notably, our study is the first to investigate the impact of pension insurance on the QOL of older adults, highlighting its significance among personal factors. Consistent with the findings of Zhang et al [18], our study incorporates HR and showed its substantial impact on QOL. We further introduce HSD, enriching the mediating pathways through which individual and family factors influence QOL. Our findings underscore the moderating effects of HR and HSD, demonstrating that the indirect effects of individual and family factors on QOL are equally important as their direct effects. Moreover, the influence on mental and physical health exhibits a similar trend across variables.

Our study found that endowment insurance plays a significant role in IF. Endowment insurance may enhance the QOL among older adults through various aspects, such as providing economic security, medical coverage, fostering social engagement, and enhancing psychological well-being [54]. Additionally, endowment insurance could potentially reduce older adults' financial dependence on their children, improve family relationships, and elevate their social status and self-esteem, thus influencing their physical and mental health and enhancing their QOL [55,56]. Our study offers valuable insights for policy makers and social welfare institutions for improving the survival and well-being of older adults.

HR is an important mediating factor for the QOL in older adults. Individual and family factors can indirectly influence the QOL of older adults by moderating HR [54]. Higher education and income levels promote easier access to nutritious food and health care services for older adults, leading to the adoption of healthier lifestyles, thus reducing their HR and enhancing their QOL [18]. Strong family relationships can provide emotional and social support for older adults, helping them cope with stress and challenges, lowering their HR and improving their QOL [57].

The impact of HSD on the QOL of older adults is relatively minor. While QOL can assist older adults in accessing necessary medical services, it cannot eliminate the influence of HR. This may be attributed to the natural decline in physiological functions as individuals age, where certain diseases or health issues may result in permanent physical damage or functional decline, thus potentially affecting the physical and mental well-being of older adults [58]. Furthermore, HSD may also be influenced by individual and family factors such as economic status and medical insurance [59]. Additionally, there may be a lag effect in the impact of HR on HSD, meaning that the influence of HR may take some time to manifest in terms of HSD [60].

In direct effect analysis, we observed the direct impacts of individual and family factors on MCS, which are significantly greater than those on PCS. This may be attributed to the multifaceted influences on MCS, including personal, social, and environmental factors [61], with these factors more likely to directly affect MCS than PCS, when not considering related intermediary effects such as health risks [62]. For instance, factors such as educational attainment and family satisfaction are more closely associated with MCS [63]. Tense family relationships, economic pressures, social discrimination, and similar factors may also contribute to MCS [64]. In contrast, PCS is more influenced by biological factors and is relatively less susceptible to direct impacts from the social environment [65]. However, incorporating HR and HSD as mediators, the impact of individual and family factors on MCS and PCS tends to converge. This could be explained by the moderating role of HR and HSD in individual and family factors and mental or physical health [66]. For instance, unhealthy dietary habits, lack of physical activity, smoking, and other health risk factors, as well as accessibility and use of medical services, which all substantially impact MCS and PCS [67]. This finding contributes to a deeper understanding of how individual and family factors affect the mental and physical health in older adults.

The subgroup analysis indicated that the influence of IF on the QOL in older adults may vary with age. This variation may be associated with increased reliance on family and social support, declining health conditions, and changes in values and life goals among older adults. First, with advancing age, older adults may experience a decline in economic and social resources, leading to increased reliance on family and social support, thereby the impact of IF on QOL declined [68,69]. Second, deteriorating health conditions in old age are characterized by a higher prevalence of chronic diseases and physical impairments, which negatively influence their QOL [70]. Although the influence of IF on health conditions may persist, the overall decline in health status might diminish the significance of IF on QOL [71]. Third, as individuals age, their values and life goals may change [72]. In the age group of 60-70 years, older adults may still have an active lifestyle and self-actualization, thus making the impact of IF on QOL more pronounced. However, in the age group of 70 years and above, older adults may prioritize family and social relationships, leading to a relatively diminished impact of IF on QOL. Consequently, to enhance the QOL among older adults, tailored policies and interventions need to be devised based on the characteristics of different age groups. For individuals aged 60-70 years, efforts can be focused on enhancing their personal capabilities and social resources, such as providing education and training and increasing employment opportunities. For those aged 70 years and older, emphasis can be placed on providing family and social support, such as establishing comprehensive older adult care systems and strengthening community-based older adult care services.

Additionally, within the context of considering comorbidity status, we found that older adults with 2 or more chronic disease conditions tend to require a greater HSD compared to those without such conditions. This may be attributed to the fact that older adults with multiple chronic diseases tend to bear a greater burden of health risks compared to those who are relatively healthy [25]. The Health Belief Model proposes that individuals are most likely to take preventive actions when they perceive a health risk as a serious threat to their own health [73]. Moreover, older adults with a greater number of chronic diseases tend to experience comparatively poorer overall physical conditions, thereby resulting in a heightened HSD [74]. Based on this, it is imperative to emphasize the cultivation of self-management awareness of comorbidities and implement additional health interventions for older adults with multiple chronic diseases. This may involve tailored health education initiatives aimed at enhancing healthy behavior and disease prevention, thereby reducing health risks and enhancing overall QOL. Furthermore, communities should develop comprehensive care service systems that cope with the varying health conditions of older adults, ultimately improving QOL.

### Strengths and Limitation

A strength of this study is the use of a large sample covering different regions of China to explore the associations between individual and family factors and QOL in older people. Furthermore, the statistical analysis was conducted using SEM based on a conceptual framework, which is an advanced statistical approach to estimate direct and indirect effects from exposure to outcome, including latent variables. This is the first study using SEM to analyze the pathways from individual and family factors to QOL via HR and HSD over the entire older adult population in China. The findings suggest potential avenues for policy and intervention to improve the QOL among older individuals.

The limitations of the study must be taken into account when interpreting the results. First, the data used in this study is from a cross-sectional survey; although the hypotheses of this study are supported by theory, experience, and statistical data, caution is still warranted in inferring causal relationships. Future research using prospective, longitudinal cohorts is needed to further validate these findings. Second, due to the constraints of the CHARLS database, there are limitations regarding the selection of variables for constructing individual and family factors, and the relevant data on the personal income data of older adults in the database are missing, so we chose per capita disposable income at the city level as a substitute, which may cause bias in the results. Third, for the physical activity data, there is a 50% missing data rate, and our sample does not include individuals with disabilities. Although sensitivity analyses indicated that the absence of physical activity data did not affect the robustness of the model, caution is advised when extrapolating conclusions to individuals with disabilities. Finally, this study included all older adults with spouses and children, caution is needed when extrapolating the conclusions to older individuals who are unmarried or without children.

### Conclusions

Both IF (education, per capita disposable income, and endowment insurance) and FF (satisfaction with a spouse and children) directly impact the QOL in older people. Meanwhile, IF and FF have equal influence on QOL through the mediating role of HR and HSD. Therefore, it is imperative to enhance social support and prioritize home care for older adults in future interventions. Enhanced access to financial and emotional support can mitigate health risks and facilitate access to quality health care, thereby improving QOL for older adults.

## Appendix

Multimedia Appendix 1 Corresponding variables in the CHARLS (China Health and Retirement Longitudinal Study) data.

Multimedia Appendix 2 Definition of variables in structural equation model.

Multimedia Appendix 3 The percentages of participants with missing data.

Multimedia Appendix 4 Correlation matrix of the variables (N=8,600).

Multimedia Appendix 5 The detailed impact pathways of IF and FF on the QOL.

Multimedia Appendix 6 Model fit indices of subgroup analysis.

Multimedia Appendix 7 The detailed impact pathways of individual factors and family factors on the quality of life in sensitivity analyses (N=4259).

Multimedia Appendix 8 Model-ft index of sensitivity analyses.

## Abbreviations

AVE: average variance extracted
AGFI: adjusted goodness of fit index
BP: bodily pain
CFA: confirmatory factor analysis
CFI: comparative fit index
CHARLS: China Health and Retirement Longitudinal Study
CR: composite reliability
CVI: content validity index
FF: family factor
GFI: goodness of fit index
GH: general health
HR: health risk
HSD: health services demand
I-CVI: item content validity index
IF: individual factor
IFI: incremental fit index
MCS: mental component summary
MH: mental health
PCS: physical component summary
PF: physical functioning
QOL: quality of life
RE: role-emotional
RMSEA: root-mean-square error of approximation
RP: role-physical
S-CVI: scale content validity index
SEM: structural equation modeling
SF: social functioning
SF-36: Short Form 36
SRMR: standardized root-mean-square residual

## Data Availability

Please contact CHARLS (China Health and Retirement Longitudinal Study) for data requests.
