# Supplementary material for: Interrelationships Among Individual Factors, Family Factors, and Quality of Life in Older Chinese Adults: Cross-Sectional Study Using Structural Equation Modeling
Source: JMIR Aging. 2024 Oct 28;7:e59818. doi: 10.2196/59818 (PMC11555452; doi:10.2196/59818)
Supplement: Multimedia Appendix 1 [file aging_v7i1e59818_app1.docx]

**Multimedia Appendix 1** Corresponding variables in the CHARLS data.

| **QOL^a^** | **CHARLS code** | |
| --- | --- | --- |
| PF^b^ | db001 db002 db003 db004 db005 db006 db007 db008 db009 |  |
| RP^c^ | db016 db017 db018 db019 db020 |  |
| BP^d^ | da041 da042s1 da042s2 da042s3 da042s4 da042s5 da042s6 da042s7 da042s8 da042s9 da042s10 da042s11 da042s12 da042s13 da042s14 da042s15 |  |
| GH^e^ | da001 da002 |  |
| VT^f^ | dc015 dc018 |  |
| SF^g^ | da056s1 da056s2 da056s3 da056s4 da056s5 da056s6 da056s7 da056s8 da056s9 da056s10 da056s11 da056s12 |  |
| RE^h^ | dc010 dc012 |  |
| MH^i^ | dc009 dc011 dc014 dc016 dc017 |  |

^a^QOL, quality of life; ^b^PF, physical functioning; ^c^RP, role-physical; ^d^BP, bodily pain; ^e^GH, general health; ^f^VT, vitality; ^g^SF, social functioning; ^h^RE, role-emotional; ^i^MH, mental health.
