# Supplementary material for: Interrelationships Among Individual Factors, Family Factors, and Quality of Life in Older Chinese Adults: Cross-Sectional Study Using Structural Equation Modeling
Source: JMIR Aging. 2024 Oct 28;7:e59818. doi: 10.2196/59818 (PMC11555452; doi:10.2196/59818)
Supplement: Multimedia Appendix 2 [file aging_v7i1e59818_app2.docx]

**Multimedia Appendix 2** Definition of variables in structural equation model.

| **Variable** | **CHARLS** **code** | **Definition** |
| --- | --- | --- |
| education | BD001_w2_4 | below secondary education”, “high school and vocational training” or “higher education” |
| endowment insurance | FN002_w3 | “Having endowment insurance” or “No endowment insurance” |
| spouse satisfaction | DC043_w3 | “Satisfied with spouse” or “Dissatisfied with spouse” |
| children satisfaction | DC044_w3 | “Satisfied with children” or “Dissatisfied with children” |
| physical activity | DA051 | “Engaging in any physical activity in the past week” or “Have not engaged in any strenuous physical activity in the past week” |
| alcohol consumption | DA067 | “Drinking alcoholic beverages in the past year” or “Not drinking alcoholic beverages in the past year” |
| unhealthy sleep | DA049 | “Sleep less than 5 hours or more than 9 hours per night” or “Sleep duration is within 6 to 8 hours” |
| siesta | DA050 | “Having a habit of taking midday naps” or “Absence of the habit of taking midday naps” |
| inpatient service | EE003 | Inpatient service utilization in the past year” or “No inpatient service utilization in the past year” |
| outpatient service | ED001 | “Received outpatient or home care services in the past month” or “Not attending outpatient or home health services in the past month” |
