# Supplementary material for: Interrelationships Among Individual Factors, Family Factors, and Quality of Life in Older Chinese Adults: Cross-Sectional Study Using Structural Equation Modeling
Source: JMIR Aging. 2024 Oct 28;7:e59818. doi: 10.2196/59818 (PMC11555452; doi:10.2196/59818)
Supplement: Multimedia Appendix 3 [file aging_v7i1e59818_app3.docx]

**Multimedia Appendix 3** The percentages of participants with missing data.

| **Characteristic** | **Count (n)** | **Missing Rate** |
| --- | --- | --- |
| age | 8600 | 1.14% |
| sex | 8600 | 0.01% |
| comorbidity | 8600 | 0% |
| education | 8600 | 0.02% |
| Per capita disposable income | 8600 | 0.77% |
| endowment insurance | 8600 | 0.58% |
| Spouse satisfaction | 8600 | 1.93% |
| children satisfaction | 8600 | 1.23% |
| alcohol consumption | 8600 | 0.05% |
| physical activity | 8600 | 50.48% |
| unhealthy sleep | 8600 | 7.97% |
| siesta | 8600 | 7.19% |
| outpatient service | 8600 | 0.14% |
| Inpatient services | 8600 | 0.14% |
