# Supplementary material for: Interrelationships Among Individual Factors, Family Factors, and Quality of Life in Older Chinese Adults: Cross-Sectional Study Using Structural Equation Modeling
Source: JMIR Aging. 2024 Oct 28;7:e59818. doi: 10.2196/59818 (PMC11555452; doi:10.2196/59818)
Supplement: Multimedia Appendix 4 [file aging_v7i1e59818_app4.docx]

**Multimedia Appendix 4** Correlation matrix of the variables (N=8,600).

| **Variables** | （1） | （2） | （3） | （4） | （5） | （6） | （7） | （8） | （9） | （10） | （11） | （12） | （13） |
| --- | --- | --- | --- | --- | --- | --- | --- | --- | --- | --- | --- | --- | --- |
| education (1) | 1 |  |  |  |  |  |  |  |  |  |  |  |  |
| per capita disposable income (2) | 0.158** | 1 |  |  |  |  |  |  |  |  |  |  |  |
| endowment insurance (3) | 0.306** | 0.256** | 1 |  |  |  |  |  |  |  |  |  |  |
| spouse satisfaction (4) | 0.033** | 0.033** | 0.052** | 1 |  |  |  |  |  |  |  |  |  |
| children satisfaction (5) | 0.012* | 0.014 | 0.034** | 0.307** | 1 |  |  |  |  |  |  |  |  |
| alcohol consumption (6) | -0.067** | 0.002 | -0.075** | -0.036** | -0.001 | 1 |  |  |  |  |  |  |  |
| physical activity (7) | -0.007 | -0.071** | -0.035** | 0.018 | 0.007 | -0.106** | 1 |  |  |  |  |  |  |
| unhealthy sleep (8) | -0.062** | -0.048** | -0.070** | -0.083** | -0.046** | 0.036** | -0.010 | 1 |  |  |  |  |  |
| siesta (9) | 0.079** | 0.074** | 0.056** | 0.035** | 0.034** | -0.059** | -0.030** | -0.096** | 1 |  |  |  |  |
| outpatient service (10) | 0.027* | -0.002 | 0.015 | -0.049** | -0.035** | 0.041** | 0.006 | 0.062** | 0.007 | 1 |  |  |  |
| inpatient service (11) | 0.009 | -0.003 | 0.029** | -0.037** | -0.037** | 0.071** | -0.098** | 0.050** | 0.030** | 0.145** | 1 |  |  |
| PCS ^a^(12) | 0.112** | 0.095** | 0.137** | 0.162** | 0.142** | -0.166** | 0.107** | -0.204** | 0.045** | -0.166** | -0.232** | 1 |  |
| MCS^b^(13) | 0.144** | 0.133** | 0.189** | 0.205** | 0.184** | -0.121** | 0.028** | -0.246** | 0.091** | -0.133** | -0.127** | 0.540** | 1 |

^a^PCS, physical component summary; ^b^MCS, mental component summary.

*P*** < .01.

*P** < .05.
