# Supplementary material for: Interrelationships Among Individual Factors, Family Factors, and Quality of Life in Older Chinese Adults: Cross-Sectional Study Using Structural Equation Modeling
Source: JMIR Aging. 2024 Oct 28;7:e59818. doi: 10.2196/59818 (PMC11555452; doi:10.2196/59818)
Supplement: Multimedia Appendix 5 [file aging_v7i1e59818_app5.docx]

**Multimedia Appendix 5** The detailed impact pathways of IF^a^ and FF^b^ on the QOL^c^.

| **pathway** | **Β^f^** | **S.E.^g^** | **C.R.^h^** |
| --- | --- | --- | --- |
| IF→HR^d^ | -0.264*** | 0.017 | -7.356 |
| FF→HR | -0.300*** | 0.026 | -7.516 |
| HR→HSD^e^ | 0.353*** | 0.075 | 6.723 |
| IF→QOL | 0.165*** | 1.499 | 5.553 |
| FF→QOL | 0.189*** | 2.233 | 5.697 |
| HR→QOL | -0.582*** | 8.225 | -7.431 |
| HSD→QOL | -0.350*** | 3.398 | -7.527 |
| IF→ endowment insurance | 0.659*** | -^i^ | - |
| IF→ per capita disposable income | 0.381*** | 909.307 | 18.817 |
| IF→ education | 0.460*** | 0.037 | 19.535 |
| FF→ spouse satisfaction | 0.588*** | - | - |
| FF→ children satisfaction | 0.523*** | 0.044 | 15.054 |
| HSD→ inpatient service | 0.418*** | - | - |
| HSD→ outpatient service | 0.347*** | 0.068 | 13.298 |
| HR→ alcohol consumption | 0.231*** | - | - |
| HR→ unhealthy sleep | 0.370*** | 0.128 | 13.025 |
| QOL→ physical component summary | 0.728*** | - | - |
| QOL→ mental component summary | 0.739*** | 0.030 | 39.287 |
| HR→ siesta | -0.146*** | 0.080 | -8.275 |
| HR→ physical activity | -0.105*** | 0.074 | -6.414 |

^a^IF, individual factors; ^b^FF, family factors; ^c^QOL, quality of life; ^d^HR, health risk; ^e^HSD, health services demand; ^f^β, standardized regression coefficient; **^g^**S.E., standard error; ^h^C.R., critical ratio; ^i^-, no applicable.

*P**** < .001.
