# Supplementary material for: Interrelationships Among Individual Factors, Family Factors, and Quality of Life in Older Chinese Adults: Cross-Sectional Study Using Structural Equation Modeling
Source: JMIR Aging. 2024 Oct 28;7:e59818. doi: 10.2196/59818 (PMC11555452; doi:10.2196/59818)
Supplement: Multimedia Appendix 6 [file aging_v7i1e59818_app6.docx]

**Multimedia Appendix 6** Model fit indices of subgroup analysis.

| Inspected Fit Indices | Acceptable Fit | Sex subgroup | Age subgroup | Comorbidity subgroup |
| --- | --- | --- | --- | --- |
| SRMR^a^ | ≤0.08 | 0.039 | 0.029 | 0.030 |
| RMSEA^b^ | ≤0.08 | 0.026 | 0.026 | 0.027 |
| GFI^c^ | >0.9 | 0.986 | 0.986 | 0.985 |
| AGFI^d^ | >0.9 | 0.977 | 0.977 | 0.976 |
| CFI^e^ | >0.9 | 0.918 | 0.922 | 0.919 |
| IFI^f^ | >0.9 | 0.918 | 0.922 | 0.919 |

^a^SRMR, standard root mean square residual; ^b^RMSEA, root-mean-square error of approximation; ^c^GFI, goodness of fit index; ^d^AGFI, adjusted goodness of fit index; ^e^CFI, comparative fit index; ^f^IFI, incremental fit index.
