# Supplementary material for: Interrelationships Among Individual Factors, Family Factors, and Quality of Life in Older Chinese Adults: Cross-Sectional Study Using Structural Equation Modeling
Source: JMIR Aging. 2024 Oct 28;7:e59818. doi: 10.2196/59818 (PMC11555452; doi:10.2196/59818)
Supplement: Multimedia Appendix 7 [file aging_v7i1e59818_app7.docx]

**Multimedia Appendix7** The detailed impact pathways of IF^a^ and FF^b^ on the QOL^c^ in sensitivity analyses (n=4,259)^j^.

| **pathway** | **β**^f^ | **S.E.^g^** | **C.R.**^h^ |
| --- | --- | --- | --- |
| IF→HR^d^ | -0.295^***^ | 0.033 | -5.774 |
| FF→HR | -0.24^***^ | 0.036 | -4.536 |
| HR→HSD^e^ | 0.314^***^ | 0.093 | 4.356 |
| HSD→QOL | -0.414^***^ | 5.136 | -6.416 |
| HR→QOL | -0.511^***^ | 9.81 | -5.338 |
| IF→QOL | 0.146^***^ | 2.677 | 3.632 |
| FF→QOL | 0.245^***^ | 2.891 | 5.897 |
| IF→Per capita disposable income | 0.378^***^ | 1558.868 | 14.706 |
| IF→education | 0.484^***^ | -^i^ | - |
| FF→ children satisfaction | 0.535^***^ | 0.068 | 10.497 |
| FF→spouses satisfaction | 0.567^***^ | - | - |
| HSD→outpatient service | 0.357^***^ | - | - |
| HSD→Inpatient services | 0.406^***^ | 0.108 | 9.698 |
| HR→alcohol consumption | 0.237^***^ | - | - |
| HR→physical activity | -0.015 (0.503) | 0.092 | -0.67 |
| HR→unhealthy sleep | 0.396^***^ | 0.197 | 8.891 |
| QOL→ physical component summary | 0.723^***^ | - | - |
| QOL→ mental component summary | 0.741^***^ | 0.044 | 27.162 |
| HR→siesta | -0.165^***^ | 0.12 | -6.091 |
| IF→endowment insurance | 0.662^***^ | 0.091 | 14.15 |

^a^IF, individual factors; ^b^FF, family factors; ^c^QOL, quality of life; ^d^HR, health risk; ^e^HSD, health services demand; ^f^β, standardized regression coefficient; **^g^**S.E., standard error; ^h^C.R., critical ratio; ^i^-, no applicable.

^j^Excluded Paticipants with no physicial activity data.

*P**** < .001.
