# Supplementary material for: Interrelationships Among Individual Factors, Family Factors, and Quality of Life in Older Chinese Adults: Cross-Sectional Study Using Structural Equation Modeling
Source: JMIR Aging. 2024 Oct 28;7:e59818. doi: 10.2196/59818 (PMC11555452; doi:10.2196/59818)
Supplement: Multimedia Appendix 8 [file aging_v7i1e59818_app8.docx]

**Multimedia Appendix8** Model-ft index of sensitivity analyses.

| Inspected Fit Indices | Acceptable Fit | model Fit Indices |
| --- | --- | --- |
| SRMR^a^ | ≤0.08 | 0.037 |
| RMSEA^b^ | ≤0.08 | 0.045 |
| GFI^c^ | >0.9 | 0.979 |
| ACFI^d^ | >0.9 | 0.967 |
| CFI^e^ | >0.9 | 0.901 |
| IFI^f^ | >0.9 | 0.901 |

^a^SRMR, standard root mean square residual; ^b^RMSEA, root-mean-square error of approximation; ^c^GFI, goodness of fit index; ^d^AGFI, adjusted goodness of fit index; ^e^CFI, comparative fit index; ^f^IFI, incremental fit index.
